# CD19 and CD20 Targeted Vectors Induce Minimal Activation of Resting B Lymphocytes

**DOI:** 10.1371/journal.pone.0079047

**Published:** 2013-11-11

**Authors:** Sabrina Kneissl, Qi Zhou, Michael Schwenkert, François-Loic Cosset, Els Verhoeyen, Christian J. Buchholz

**Affiliations:** 1 Molecular Biotechnology and Gene Therapy, Paul-Ehrlich-Institut, Langen, Germany; 2 Department of Biology, University of Erlangen-Nuremberg, Erlangen, Germany; 3 CIRI, International Center for Infectiology Research, EVIR team, Inserm U1111, CNRS, UMR5308, Université de Lyon-1, ENS de Lyon, Lyon, France; 4 INSERM, U895, Centre de Médecine Moléculaire (C3M), équipe 3, Nice, France; Karolinska Institutet, Sweden

## Abstract

B lymphocytes are an important cell population of the immune system. However, until recently it was not possible to transduce resting B lymphocytes with retro- or lentiviral vectors, making them unsusceptible for genetic manipulations by these vectors. Lately, we demonstrated that lentiviral vectors pseudotyped with modified measles virus (MV) glycoproteins hemagglutinin, responsible for receptor recognition, and fusion protein were able to overcome this transduction block. They use either the natural MV receptors, CD46 and signaling lymphocyte activation molecule (SLAM), for cell entry (MV-LV) or the vector particles were further modified to selectively enter via the CD20 molecule, which is exclusively expressed on B lymphocytes (CD20-LV). It has been shown previously that transduction by MV-LV does not induce B lymphocyte activation. However, if this is also true for CD20-LV is still unknown. Here, we generated a vector specific for another B lymphocyte marker, CD19, and compared its ability to transduce resting B lymphocytes with CD20-LV. The vector (CD19ds-LV) was able to stably transduce unstimulated B lymphocytes, albeit with a reduced efficiency of about 10% compared to CD20-LV, which transduced about 30% of the cells. Since CD20 as well as CD19 are closely linked to the B lymphocyte activation pathway, we investigated if engagement of CD20 or CD19 molecules by the vector particles induces activating stimuli in resting B lymphocytes. Although, activation of B lymphocytes often involves calcium influx, we did not detect elevated calcium levels. However, the activation marker CD71 was substantially up-regulated upon CD20-LV transduction and most importantly, B lymphocytes transduced with CD20-LV or CD19ds-LV entered the G1b phase of cell cycle, whereas untransduced or MV-LV transduced B lymphocytes remained in G0. Hence, CD20 and CD19 targeting vectors induce activating stimuli in resting B lymphocytes, which most likely renders them susceptible for lentiviral vector transduction.

## Introduction

Playing a major role in the humoral immune response B lymphocytes are responsible for antibody production, perform the role of antigen-presenting cells (APCs) and eventually mature into memory B lymphocytes after activation via antigen binding. These properties make B lymphocytes important target cells for immunotherapy approaches [1] and to investigate basic questions in B lymphocyte related immunology. Furthermore, they are target cells in many lymphomas like non-Hodgkins or Burkitt lymphoma. However, until recently it was not possible to efficiently transduce resting B lymphocytes with retro- or lentiviral vectors making them unsusceptible for stable genetic manipulations or gene therapy approaches [2]. Although lentiviral vectors can transduce many types of non-proliferating cells, primary human lymphocytes normally require stimulation with cytokines or other factors and entry from G0 into G1b phase of cell cycle to become transduced by these vectors. This holds true also for lentiviral vectors pseudotyped with the glycoprotein of the vesicular stomatitis virus (VSVG), which are basically the “gold standard” to which all other lentiviral pseudotypes are compared. Using different lentiviral pseudotypes it was observed that in resting lymphocytes post-entry steps like completion of reverse transcription, nuclear import and chromosomal integration of the transgene do not occur [2]–[4].

However, recently we demonstrated that lentiviral vectors pseudotyped with modified measles virus (MV) envelope proteins hemagglutinin (H), responsible for receptor recognition, and fusion (F) protein are able to transduce resting B and T lymphocytes with high efficiency [5]–[7]. The MV glycoproteins that are derived from the NSe variant of the MV vaccine strain Edmonston B, are truncated in their cytoplasmic tails to allow efficient incorporation into the lentiviral envelope. Thereupon, the respective vector particles (MV-LV) are able to mediate cell entry via the native MV receptors human CD46 and signaling lymphocyte activation molecule (SLAM). We found that interaction with both receptors is essential to facilitate efficient transduction of resting lymphocytes [8], [9]. The recently identified third MV receptor nectin-4 does not play a role in lymphocyte transduction as it is not expressed on this cell type [10], [11]. Importantly, MV-LV transduction does not lead to lymphocyte activation as the cells remain in G0 state of cell cycle [5], [6].

MV glycoprotein pseudotyping can also be utilized to generate targeted vectors that mediate specific entry into cell types of choice. To confer specific target cell entry to the MV-LVs, we abolished recognition of the MV receptors CD46 and SLAM by mutating the truncated H protein at four residues in its ectodomain, namely Y481A, R533A, S548L and F549S [12]. The desired receptor specificity is provided by displaying a single-chain antibody (scFv) specific for the target receptor on the mutated H protein (H_mut_-scFv). Using this strategy, very different cell surface molecules including type1-membrane glycoproteins (CD105), pentaspan membrane glycoproteins (CD133), membrane tetraspan calcium channels (CD20) as well as multi-subunit ion-channels (glutamate receptors, GluR) can be used for cell specific entry by these vectors. The respective targeting vectors were not only able to selectively transduce receptor-positive cell lines, but also the corresponding target receptor-positive primary cells [7], [13]. More importantly, remarkable target specificity was observed *in vivo*, upon local or systemic injection into mice [13]–[16].

Using CD20, a marker exclusively expressed on B lymphocytes, as entry receptor, we found that the respective vector (CD20-LV) is able to efficiently transduce unstimulated, resting primary human B lymphocytes [7], [17]. Since CD20 is especially expressed on differentiated B cells but not on B cell precursors such as pro-B and pre-B cells [18], here we generated a B lymphocyte targeted vector utilizing CD19 for cell entry, which is a specific marker for all B cell populations. As both, CD20 and CD19, are closely linked to the B lymphocyte activation pathway [19]–[22], we investigated if engagement and cross-linking of the receptors by the corresponding targeted vector particles induces activating stimuli in resting B lymphocytes, thereby rendering them susceptible for transduction. We found that the CD19-targeted LV was highly specific for CD19^+^ cells in cell mixtures and stably transduced unstimulated primary human B lymphocytes, albeit with a reduced efficiency compared to CD20-LV. The activation marker CD71 was substantially up-regulated on unstimulated B lymphocytes upon transduction with CD20-LV. Furthermore, CD20-LV and CD19ds-LV transduced B lymphocytes entered into the G1b cell cycle phase.

## Results

### Generation of CD19 Targeting Vectors

**Figure 1:**
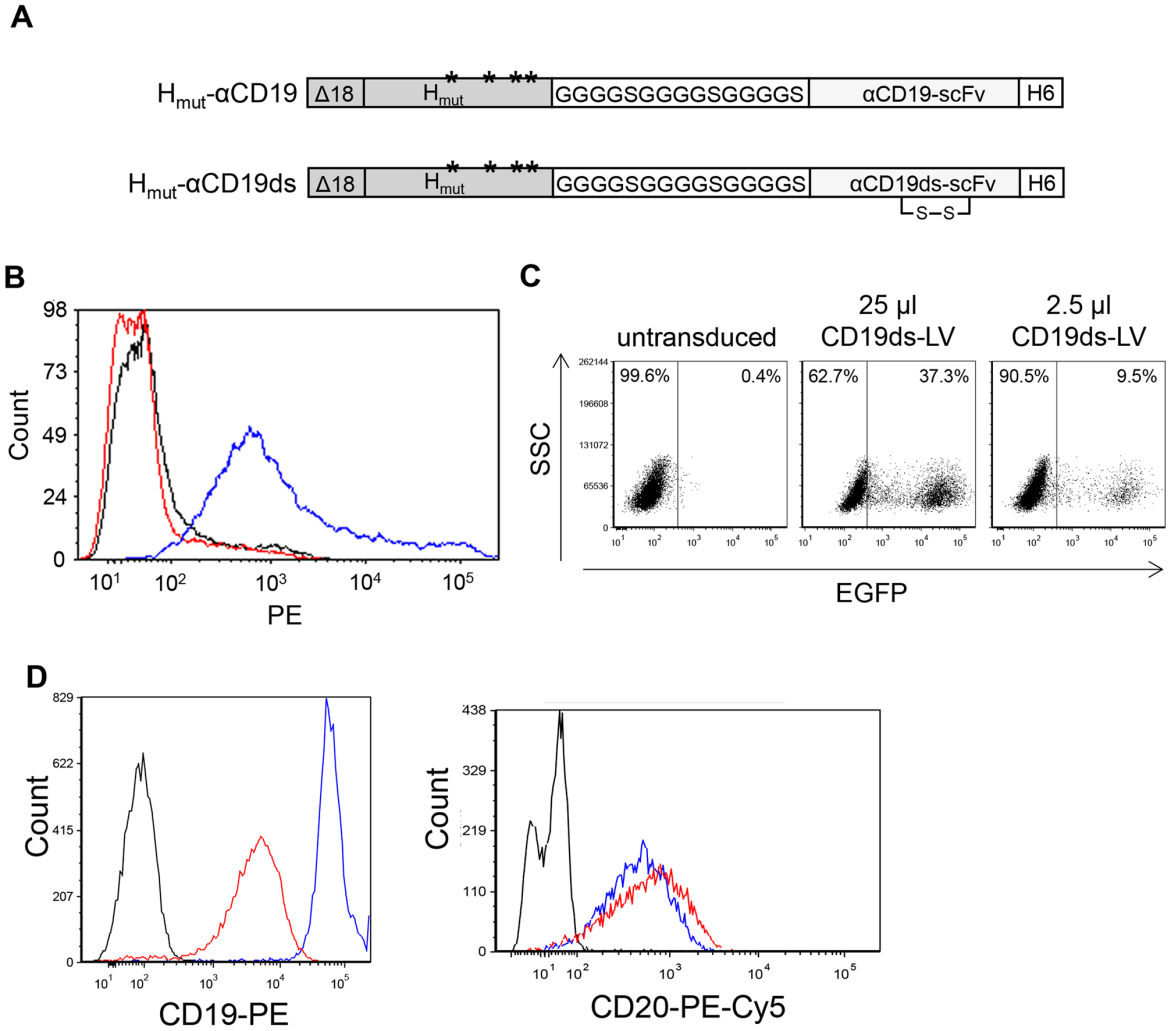
A stabilizing disulfide bond in αCD19ds-scFv is essential for generating CD19 targeting vectors. (**A**) Schematic drawing of the cytoplasmic tail-truncated hemagglutinin envelope proteins displaying two αCD19 single-chain antibody (scFv) variants. In the mutated hemagglutinin protein (H_mut_) that is derived from the NSe variant of the measles virus (MV) vaccine strain Edmonston B, mutations in the MV receptor recognition regions Y481A, R533A, S548L and F549S (ectodomain) [12] are indicated by asterisks. A glycine-serine linker ((G_4_S)_3_) is used as linker region between H_mut_ and scFv. The αCD19-scFv and αCD19ds-scFv differ in the presence of a disulfide bond in αCD19ds-scFv. A histidine tag (H6) is present at the scFv C-terminus. Both hemagglutinin proteins are truncated by 18 amino acids in their cytoplasmic tail (Δ18) to allow incorporation into the lentiviral envelope. (**B**) Cell surface transport of the two different H variants was investigated upon transfection of plasmids pCG-H_mut_-αCD19 (red line) and pCG-H_mut_-αCD19ds (blue line) into HEK-293T cells. The empty expression plasmid pCG-1 (black line) was used as control. Forty-eight hours after transfection, the cells were stained with a PE-conjugated anti-His antibody to detect cell surface expression of the H constructs by FACS analysis. The respective histograms are shown. (**C**) To determine the titer of vector particles pseudotyped with H_mut_-αCD19ds (CD19ds-LV), CD19^+^ Raji cells were transduced with serial dilutions of the concentrated vector particles. After 72 h, the percentage of EGFP^+^ cells was determined by FACS analysis. (**D**) To determine cell surface down-regulation of CD19 after CD19ds-LV transduction, CD19^+^/CD20^+^ Raji cells were transduced with CD19ds-LV vectors at an MOI of 2 or remained untransduced. Forty-eight hours later, untransduced cells were stained with PE-conjugated anti-CD19 (**left**; blue line) and PE-Cy5-conjugated anti-CD20 (**right**; blue line) antibody, or with the respective isotype controls (black lines). CD19ds-LV transduced cells were stained with PE-conjugated anti-CD19 (**left**; red line) and PE-Cy5-conjugated anti-CD20 (**right**; red line) antibody. Then, CD19 and CD20 cell surface expression was analyzed by FACS.

For the generation of CD19 targeting vectors the genetic information for two variants of the CD19-specific scFv (CD19-scFv and CD19ds-scFv) [23], [24] was fused to the H_mut_ reading frame (**Figure 1A**). In pH_mut_-αCD19ds the CD19ds-scFv was stabilized by a disulfide bond through introduction of cysteine residues into the conserved framework regions [25]. For incorporation into lentiviral vector particles, which bud from the cell surface, it is essential that the H constructs are expressed on the surface of HEK-293T packaging cells. This was the case only for the disulfide-stabilized H_mut_-αCD19ds protein (**Figure 1B**). Hence, this construct was used for all further experiments. After expressing H_mut_-αCD19ds protein together with all other components of a lentiviral vector in HEK-293T cells, concentrated CD19 targeting vectors (CD19ds-LV) with a titer of 4.0×10^6^ transducing units (t.u.)/ml could be generated (**Figure 1C**).

**Figure 2:**
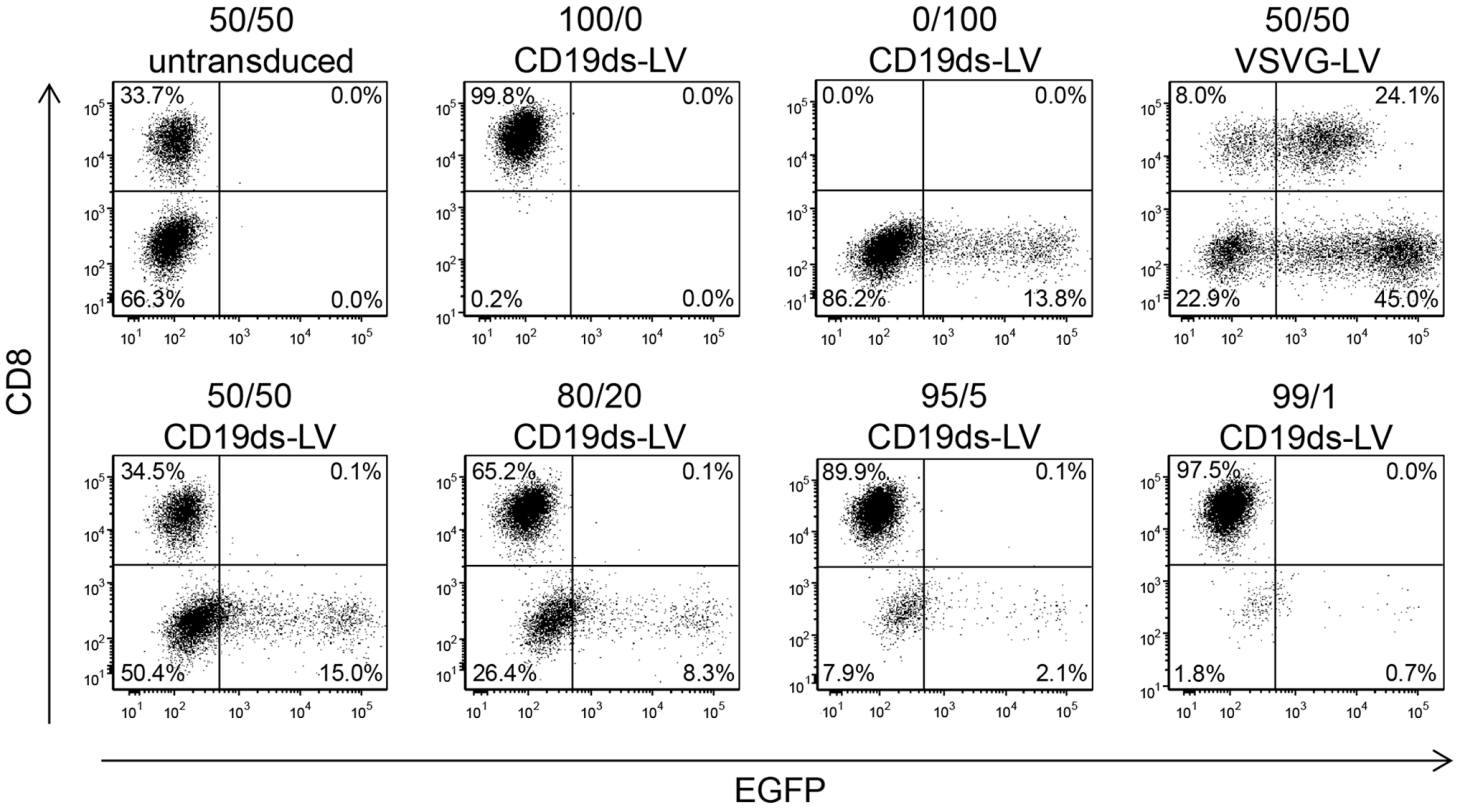
In presence of high numbers of non-target cells CD19ds-LV is specific for CD19^+^ cells. CD19^−/^CD8^+^ Molt 4.8 cells were mixed in the indicated ratios with CD19^+^/CD8^−^ Raji cells and transduced with CD19ds-LV or VSVG-LV vector particles at an MOI of 2 (refers to the total cell number). After 72 h, the cells were stained for CD8 expression and the percentage of EGFP^+^/CD8^−^ cells was determined by FACS analysis. A gating strategy was chosen in which the untransduced cells lay well within the EGFP-negative quadrants as shown in the upper left diagram.

To evaluate the specificity of CD19ds-LV, we transduced a series of mixtures of CD19^+^ Raji cells and CD19^−^ Molt 4.8 cells. Besides by their CD19 levels, Molt 4.8 cells (CD8^+^/CD19^−^) can also be distinguished from Raji cells (CD8^−/^CD19^+^) by CD8 expression. Since we observed that CD19 was strongly down-regulated upon CD19ds-LV transduction (**Figure 1D**), we chose CD8 as cell discriminating marker in this experiment. Different ratios of Raji and Molt 4.8 cells were incubated with CD19ds-LV or VSVG-LV control vectors, transferring the gene for the enhanced green fluorescent protein (EGFP). 72 h later, the cells were stained for CD8 and analyzed by FACS. CD19ds-LV vectors selectively transduced the CD8^−^ target cell population even when it made up only 1% of the cell mixture, whereas non-target cells remained untransduced (**Figure 2**). Normalized to the total number of CD8^−/^CD19^+^ cells, CD19ds-LV transduced about 20–30% of target cells in each cell mixture. In contrast, VSVG-LV did not discriminate both cell lines (**Figure 2**). Hence, CD19ds-LV demonstrated a high specificity for its target cell population.

### Transduction of Unstimulated Primary Human B Lymphocytes by CD20-LV and CD19ds-LV

**Figure 3:**
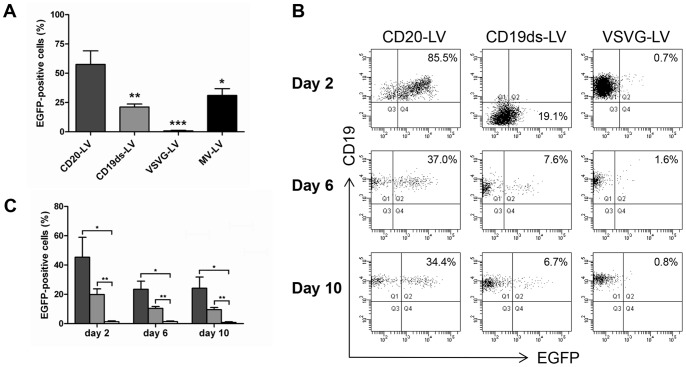
Stable transduction of unstimulated primary human B lymphocytes by CD20-LV and CD19ds-LV. (**A**) Freshly isolated primary human B lymphocytes from six different donors were transduced with CD20-LV, CD19ds-LV or MV-LV at an MOI of 2, or VSVG-LV at an MOI of 100. Forty-eight hours later, B lymphocytes were identified by their CD20 expression and the percentage of EGFP^+^ cells in the CD20-gated cells was determined by FACS analysis. Results are expressed as mean ± SEM. *, ** and *** indicate *P*<0.05, *P*<0.01 and *P*<0.001 versus transduction with CD20-LV, respectively. Data were analyzed by one-way ANOVA. (**B–C**) To verify stable integration of the *egfp* gene into the B lymphocyte genome, freshly isolated primary human B lymphocytes (purity was ∼99.8%) from three different donors were transduced with the indicated vectors. Transduced cells were cultivated in presence of 10 ng/ml rhIL-15 and 10 ng/ml rhIL-2 for 10 days on MS-5 feeder cells. The percentage of CD19^+^/EGFP^+^ cells in the CD20-gated cells was determined by FACS analysis at the indicated time points. (**B**) FACS blots of one representative experiment are shown (at least 2000 B lymphocytes were gated for analysis). (**C**) Diagram showing a summary of all three independent experiments. Dark gray column: CD20-LV; light gray column: CD19ds-LV; white column: VSVG-LV. Results are expressed as mean ± SEM. * and ** indicate *P*<0.05 and *P*<0.01 for CD20-LV and CD19ds-LV versus VSVG-LV, respectively. Data were analyzed by Student’s t-test without adjustment of multiple comparisons.

We have previously shown that lentiviral vectors specific for the B lymphocyte marker CD20 (CD20-LV) efficiently transduce unstimulated primary human B lymphocytes [7]. Now, we investigated if also CD19ds-LV vectors are able to transduce this kind of cells. For this purpose, freshly isolated primary human B lymphocytes were incubated with CD20-LV, CD19ds-LV, VSVG-LV or MV-LV in complete absence of activating cytokines. Forty-eight hours after incubation with CD20-LV on average about 60% of unstimulated B lymphocytes of six different donors were EGFP^+^, whereas with VSVG-LV as a negative control no EGFP^+^ cells were detectable. MV-LV as a positive control mediated EGFP expression in about 40% of unstimulated B cells (**Figure 3A**). Also CD19ds-LV incubation led to EGFP^+^ B lymphocytes, albeit with a lower efficiency of about 20% (**Figure 3A**). To assess the stability of gene transfer, we continued to culture the transduced B lymphocytes of three different donors on an MS-5 feeder cell layer in presence of rhIL-15 and rhIL-2 for up to 10 days. After an initial decrease in EGFP^+^ cells during the first 4 days, which might be due to EGFP protein transfer, CD20-LV transduced cells remained at a constant level of about 30% EGFP expressing cells (**Figure 3B,C**). Upon CD19ds-LV transduction, the percentage of EGFP^+^ cells also slightly decreased from day 2 to day 6, but then the level of EGFP expressing cells was stable (**Figure 3B,C**). This data confirm the stable transgene expression mediated by CD20-LV and CD19ds-LV transduction. Moreover, during the whole observation period, the percentages of EGFP^+^ cells for CD19ds-LV transduced cells were significantly higher than that of VSVG-LV transduced cells (**Figure 3C**). Interestingly, CD19ds-LV transduction also strongly and temporarily down-regulated CD19 expression in primary human B lymphocytes before the CD19 level was recovered six days after transduction (**Figure 3B**). In summary, these data demonstrate that in addition to CD20-LV, also CD19ds-LV transduced unstimulated primary human B lymphocytes.

### CD20-LV and CD19ds-LV Induce Minimal Activation of Resting Primary Human B Lymphocytes

**Figure 4:**
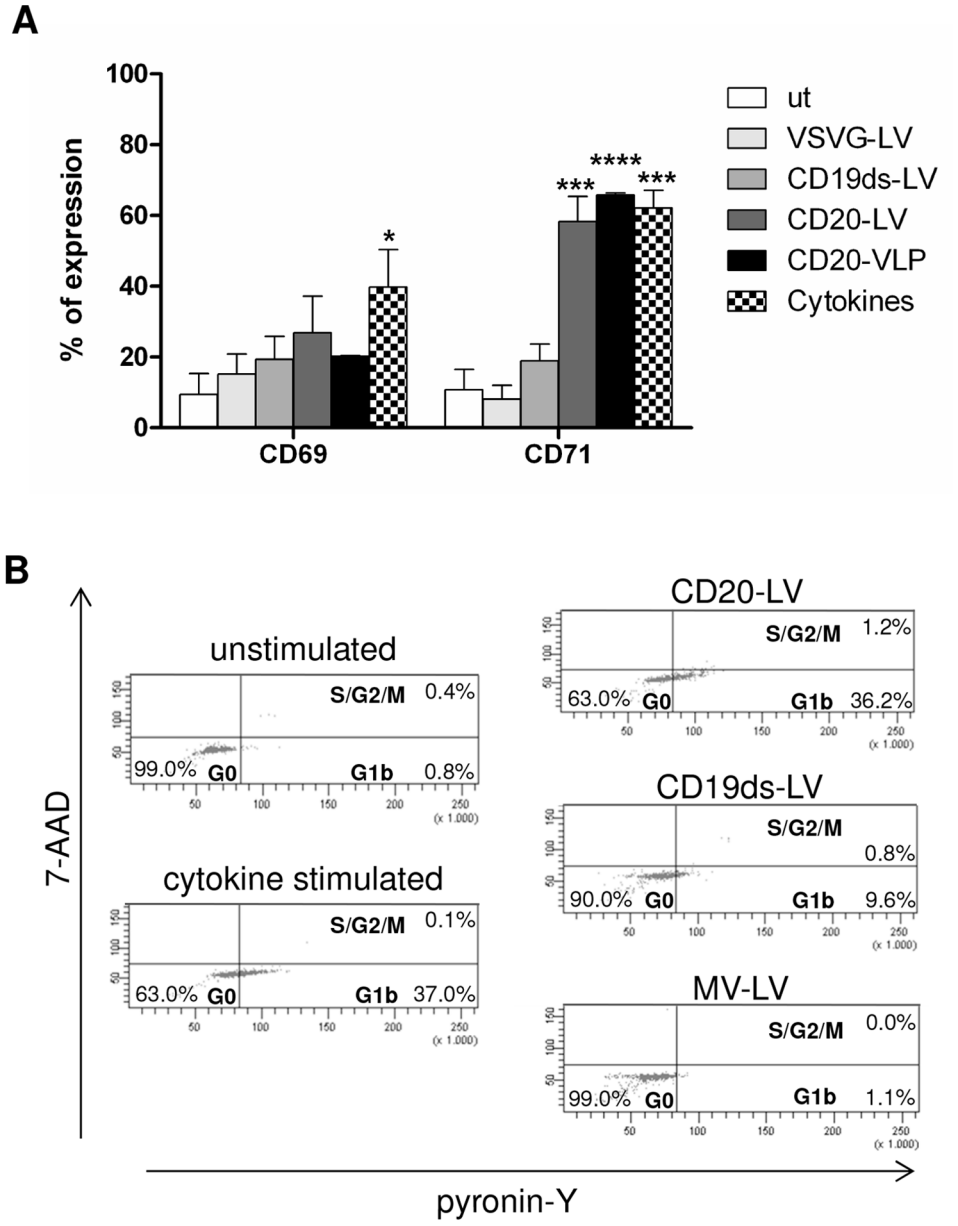
CD20-LV and CD19ds-LV induce minimal activation of resting primary human B lymphocytes. (**A**) Freshly isolated primary human B lymphocytes were left untransduced or were incubated with CD20-LV, CD19ds-LV or VSVG-LV at an MOI of 1.5–2 or with CD20-VLP at the same HIV p24 value as CD20-LV. As positive control, B lymphocytes were activated with a cytokine cocktail consisting of 50 ng/ml IL-2, 300 ng/ml CD40Ligand, 10 ng/ml IL-4 and 10 ng/ml IL-10. Forty-eight hours later, the cell surface expression of the activation markers CD69 and CD71 was determined by FACS analysis using appropriate antibodies. Mean values of CD69^+^ and CD71^+^ cells, respectively, of four different donors and SEM are shown. *, ***, and ****indicate *P*<0.05, *P*<0.001 and *P*<0.0001 versus untransduced cells, respectively. Data were analyzed by two-way ANOVA. (**B**) Freshly isolated primary human B lymphocytes were transduced by CD20-LV (MOI 3), CD19ds-LV (MOI 3) or MV-LV (MOI 5) vectors or remained untransduced. As positive control, B lymphocytes were activated with the same cytokine cocktail as described for (A). Forty-eight hours later, cell cycle progression was monitored by simultaneously visualizing the RNA (pyronin-Y) and DNA (7-AAD) content of the B lymphocytes by FACS analysis. The percentages of cells in G0, G1b or S/G2/M phase of the cell cycle are indicated. One representative experiment out of four independent experiments is shown.

Since CD20 as well as CD19 are closely linked to the B lymphocyte activation pathway [19]–[22], we hypothesized that the efficient transduction of resting B lymphocytes by CD20-LV and CD19ds-LV was due to the induction of activating stimuli in B lymphocytes upon vector engagement. Therefore, we investigated if the activation markers CD69 and CD71 were up-regulated on primary human B lymphocytes 48 h after transduction with CD20-LV, CD19ds-LV or VSVG-LV. As controls, untransduced B lymphocytes and cytokine stimulated B lymphocytes were included. Although both CD19ds-LV and CD20-LV show a tendency to up-regulate early (CD69) and late (CD71) activation markers [26], only CD20-LV up-regulated CD71 in a similar range as cytokine treatment (**Figure 4A**). Also CD20-targeted virus like particles (CD20-VLP) induced marker up-regulation to a similar extent as CD20-LV. As expected, no difference between VSVG-LV treated or untreated cells was observed (**Figure 4A**). Furthermore, some of the CD20-LV and CD19ds-LV transduced B lymphocytes of four different donors progressed from the G0 to the G1b phase of the cell cycle (**Figure 4B** **and** **Table 1**). For example, 48 h after transduction 36.2% of CD20-LV and 9.6% of CD19ds-LV transduced cells resided in G1b, whereas untransduced cells remained completely in the G0 phase (**Figure 4B** **, Donor 1 in** **Table 1**). Surprisingly, for two donors CD20-LV transduced cells entered the cell cycle as efficiently as B lymphocytes that were stimulated by cytokine incubation (**Table 1**). In general, CD20-LV induced more pronounced cell cycle progression and higher expression levels of both activation markers than CD19ds-LV in all of the four donors tested, although there was some variability between the donors in the four independent cell cycle experiments (**Table 1** and **Figure 4A**). As published previously [6], MV-LV vectors did not induce cell cycle progression of resting B lymphocytes (**Figure 4B** **and** **Table 1**), confirming the validity of our assay. Notably, for the cell cycle experiments B lymphocytes isolated instantly after blood donation using the BD Vacutainer™ CPT™ system were used. This likely explains the lower B lymphocyte transduction rates as compared to those seen with B lymphocytes derived from buffy coats by centrifugation over Histopaque-1077 (**Table 1** **and** **Figure 3A**).

**Table 1:** Cell cycle progression of resting primary human B lymphocytes after transduction with targeted vectors.^1.^

	Donor 1			Donor 2			Donor 3			Donor 4		
	G1bcells [%]	GFP^+^cells [%]	MOI	G1bcells [%]	GFP^+^cells [%]	MOI	G1bcells [%]	GFP^+^cells [%]	MOI	G1bcells [%]	GFP^+^cells [%]	MOI
CD20-LV	36.2	13.5	3	5.9	7.4	2	9.0	38.0	2	10.0	59.0	2
CD19ds-LV	9.6	4.9	3	5.1	25.8	2	2.8	16.0	2	n.d.^5^	n.d.	n.d.
MV-LV	1.1	11.8	5	2.0	46.4	5	0.9	35.0	2	0.0	40.0	10
untransduced^2^	0.8	n.a.^4^	n.a.	1.6	n.a.	n.a.	0.8	n.a.	n.a.	1.0	n.a.	n.a.
untransduced^3^	37.0	n.a.	n.a.	14.6	n.a.	n.a.	49.0	n.a.	n.a.	10.0	n.a.	n.a.

1 Cells were analyzed 48 h after transduction.

2 Unstimulated B lymphocytes were analyzed.

3 B lymphocytes were stimulated for 48 h with a cytokine cocktail consisting of 50 ng/ml IL-2, 300 ng/ml CD40Ligand, 10 ng/ml IL-4 and 10 ng/ml IL-10.

4 not applicable.

5 not done.

### CD20-VLP and CD19ds-VLP do not Induce Measurable Calcium Influx into Resting B Lymphocytes

**Figure 5:**
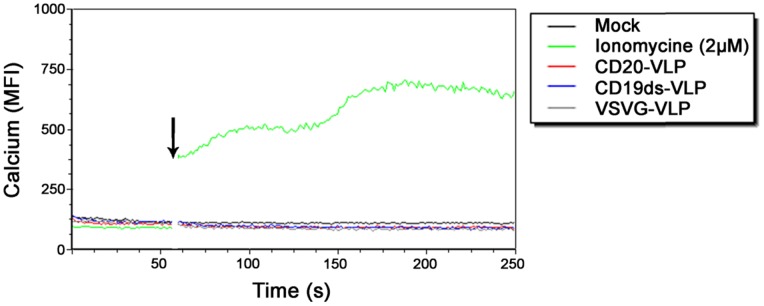
CD20-VLP and CD19ds-VLP do not induce calcium influx into unstimulated primary human B lymphocytes. Freshly isolated primary human B lymphocytes were labeled with Calcium Sensor Dye eFluor® 514 and labeled cells were assessed by flow cytometry to establish a baseline level of fluorescence. Then cells were removed, incubated with the indicated stimulants, and replaced immediately for further flow cytometric analysis. The transient increase in intracellular calcium concentration was recorded by monitoring the change in MFI of the cells. To determine the background auto-fluorescence of the cells, one sample was recorded without the addition of any stimulant after baseline monitoring (mock). The gap in the histogram reflects the time-period when the tube containing the cells was removed from the instrument to add the stimulants. The arrow indicates the time-point of addition of the stimulant. VLP: virus like particles; MFI: mean fluorescence intensity.

It has been shown that certain types of CD20- and CD19-specific monoclonal antibodies can cause calcium influx into B cell lines [22], [27]. Moreover, the sustained elevation of cytoplasmic free calcium achieved through a combination of its release from intracellular stores and influx of extracellular calcium via membrane channels, is known to be required for B cell activation [28]. Since the targeting domains of CD20-LV and CD19ds-LV were monoclonal antibody derived and both of these two vectors induced B cell activation, we next investigated if transduction of resting B lymphocytes with CD20-LV and CD19ds-LV, respectively, induced calcium influx. To avoid any interference with GFP pseudotransduction in the calcium influx assay, we generated VLPs instead of lentiviral vectors. CD20-VLP, CD19ds-VLP and VSVG-VLP were equipped with the same envelope as the corresponding lentiviral vector, but contained no transfer vector. The same amounts of CD20-VLP, CD19ds-VLP and VSVG-VLP (normalized by p24 titer) were added to eFluor® 514-labeled resting primary human B lymphocytes. Calcium influx was monitored by changes in the mean fluorescence intensity (MFI) of the eFluor® 514-labled B lymphocytes that are directly related to changes in the cytoplasmic calcium concentration. As a positive control, B lymphocytes treated with 2 µM ionomycine were included. While ionomycine induced significant calcium influx, neither CD20-VLP, CD19ds-VLP nor VSVG-VLP induced any detectable calcium influx (**Figure 5**). It thus appears that the association of the displayed scFvs on the surface of CD20-LV and CD19ds-LV with their receptors CD20 and CD19 does not induce calcium influx into resting primary human B lymphocytes.

## Discussion

Although lentiviral vectors can transduce many types of non-proliferating cells, primary human lymphocytes normally require stimulation and entry from G0 into G1b phase of the cell cycle to become transduced by these vectors [2]–[4]. However, recently we demonstrated that lentiviral vectors pseudotyped with modified MV glycoproteins are able to transduce resting primary human lymphocytes [5]–[7]. These vectors use either the natural MV receptors CD46 and SLAM for cell entry (MV-LV) or the B lymphocyte marker CD20 (CD20-LV). Here, we show in addition that unstimulated B lymphocytes can be transduced via CD19, another B lymphocyte specific molecule.

It has been shown that MV-LV vectors do not activate B lymphocytes via the transduction process and that simultaneous contact to both MV receptors CD46 and SLAM is crucial [6], [8], [9]. We proposed that transduction is facilitated by downstream signaling from the MV receptors leading to actin cytoskeleton remodeling [17]. Remodeling of the cortical actin cytoskeleton has been shown to be a prerequisite for productive entry of HIV-1 into resting lymphocytes [29]. These cytoskeleton rearrangements might permit transport of vector particles and reverse transcribed DNA to the nucleus, subsequently allowing gene integration [9], [17].

In addition, CD20-LV vectors, which use CD20 for cell entry instead of CD46 and SLAM, also efficiently transduce resting B lymphocytes. Since CD20 is involved in B lymphocyte activation [19]–[22], we suggested that the transduction mechanism of CD20-LV was different from that of MV-LV. Minimal activation of the cells into G1b phase of cell cycle after engagement and cross-linking of CD20 by CD20-LV vector particles might be the main factor to allow transduction of resting B lymphocytes [17]. To address this question, here we generated a novel B lymphocyte specific lentiviral vector via targeting to CD19.

CD19 is an essential co-receptor on the surface of B lymphocytes. It is part of a complex also containing CD21, CD81 and leu13. However, there is increasing evidence that CD19 may also be present and function alone, independently of the CD21-CD19-CD81-leu13 complex [19]. It has been proposed to function as an adaptor-like protein, mediating the recruitment and activation of signaling molecules, like phosphatidylinositol 3-kinase (PI3K), to B cell receptor (BCR) microclusters [30], [31]. Furthermore, it might stabilize and amplify signaling within individual BCR microclusters, thus enhancing BCR signaling and facilitating B cell activation [19].

For CD20, there is increasing evidence that it has a function in BCR induced calcium entry [21], [22], [32]. Intracellular signaling events that follow BCR ligation, lead to rapid release of calcium from the endoplasmic reticulum into the cytoplasm. Depletion of the intracellular calcium stores then triggers a second phase of calcium mobilization, characterized by sustained calcium entry into the cell via plasma membrane-associated “store-operated” calcium entry (SOCE) channels. The amplitude and duration of the calcium response defines gene expression by calcium-sensitive proteins [32]. It has been shown that BCR-activated calcium entry was reduced after siRNA-mediated down-regulation of CD20 in a human B cell line [33] and in CD20-deficient murine B cells [34]. This firmly points to a role for CD20 in BCR-activated SOCE, although it is not yet clear whether CD20 is a channel, a component of a channel, or if it regulates SOCE in some other ways [21], [32]. The binding of most mAbs to CD20 leads to its redistribution into lipid rafts and generates transmembrane signals that result in enhanced phosphorylation of CD20 and e.g. induction of c-myc and B-myb oncogene expression. Furthermore, it has been shown that the CD20 mAb 1F5 activates dense resting (G0) tonsillar B cells and drives them into the G1 stage of the cell cycle. However, although binding of the 1F5 mAb to CD20 can deliver an activation signal, it is insufficient on its own to initiate entry into the S phase of the cell cycle and subsequent clonal division [21], [35].

In this study, unstimulated primary human B lymphocytes were transduced by both CD20-LV and CD19ds-LV, the latter being generally less efficient. Different reasons may account for this: There is a 4-fold higher CD20 than CD19 expression level on the surface of B lymphocytes [36] and we know from previous work that target receptor density is crucial for efficient transduction [15]. We also observed a strong down- regulation of CD19 surface expression after transduction with CD19ds-LV, suggesting endocytosis of CD19 after vector engagement. As the MV glycoproteins are functional under neutral pH only, such particles will be unable to escape from the endosomes and deliver the transferred gene to the cell nucleus. Moreover, up-regulation of activation markers was found to be more pronounced with CD20-LV than with CD19ds-LV.

Our cell cycle analysis revealed that cells transduced by targeted vectors entered the G1b phase, whereas untransduced cells or cells transduced with MV-LV remained in G0. Especially, CD20-LV transduced B lymphocytes entered the cell cycle comparably efficient as cytokine stimulated cells in two out of four donors tested. This is in line with the published results for 1F5 antibody binding to CD20, which also induces cell cycle entry into G1, but not entry into the S phase [21], [35]. Thus, binding of CD20 by αCD20-scFv displayed on the vector particle surface has the same effect as antibody binding and it has been shown in numerous publications that binding of antibodies to CD20 induces intracellular signals [21], [28]. Activation marker expression and cell cycle entry was less pronounced for CD19ds-LV. Hence, binding of CD19 by CD19ds-LV did not induce such strong activating signals as CD20 binding. This is in line with many publications describing the stimulation of B lymphocytes by CD20-specific antibodies while such data are absent for CD19-specific antibodies [21], [35]. This may also well explain the lower transduction efficiency reached with CD19ds-LV on unstimulated B lymphocytes as compared to CD20-LV.

Neither vector entry via CD20 nor via CD19 induced a measurable Ca^2+^ flux into B lymphocytes, whereas the ionomycine positive control increased Ca^2+^ flux about six fold above the baseline level demonstrating that the sensitivity of our assay was comparable to that of published flow cytometry based calcium assays [37], [38]. The absence of Ca^2+^ influx after CD20-VLP and CD19ds-VLP incubation was unexpected as CD19 as well as CD20 are believed to play a role in BCR induced B lymphocyte activation, which also involves Ca^2+^ mobilization [20], [21]. Especially CD20 seems to be involved in BCR mediated Ca^2+^ entry [21], [22], [32]. It has been published that hyper-cross-linking of bound CD20 mAb induces Ca^2+^ influx into B cell lines [22]. However, not all CD20-specific antibodies induce calcium flux after hyper-cross-linking. CD20 mAbs can be separated into two distinct groups, whereby Type I mAbs are defined by their ability to redistribute CD20 into detergent-insoluble plasma membrane domains and to evoke complement-dependent cellular cytotoxicity (CDC). Only Type I mAbs are able to induce calcium flux after hyper-cross-linking with anti-Fc reagents [22].

As there are many scFvs presented on the vector particle surface, it is likely, that CD20 molecules are cross-linked by bound vector particles. However, this might not reflect the same situation as hyper-cross-linked antibodies. Furthermore, it is not known if the antibody clone B9E9 from which the presented CD20-scFv is derived [39] belongs to Type I anti-CD20 antibodies. In general it has been shown that binding of different CD20 antibodies leads to different effects in B lymphocytes, probably because they bind different epitopes on CD20 [21], [35]. Binding by the displayed αCD20-scFv seems to induce entry into G1b phase of cell cycle without inducing detectable Ca^2+^ influx. For CD19, although on mouse B lymphocytes the CD19 engagement by dimeric anti-CD19 mAb induces [Ca^2+^]_i_ responses, it is suggested that the extent and valency of CD19 ligation determine the regulatory function of CD19 on B cell activation and regulate the BCR-induced [Ca^2+^]_i_ [40]. Possibly, the affinity of the αCD19ds-scFv or the number of displayed αCD19ds-scFv on vector particles is not sufficient to trigger calcium responses. The absence of any detectable induction of Ca^2+^ influx is in line with no increase in CD19ds-LV mediated transduction, when we activated B lymphocytes of three different donors with CD20-LV before adding CD19ds-LV (data not shown). This reflects that although a minimal B lymphocyte activation is critical for transduction, signals induced by the interaction between the CD20-scFv and CD20 are only active in cis but not in trans. Possibly, this interaction opens an efficient vector entry route for particles bound to CD20 but not for particles bound to other cell surface receptors.

In conclusion, we demonstrated that CD20 and CD19 targeting vectors specifically transduce unstimulated B lymphocytes, probably by inducing minimal activation of the cells into G1b phase of cell cycle. Hence, retargeted vectors are not only able to mediate specific gene transfer, but also modulate cell physiology.

## Materials and Methods

### Ethics Statement

The research has been approved by the Ethics Committee of the University Hospital Frankfurt, Germany. Written informed consent was obtained from each donor.

### Plasmid Constructions

The plasmid pCG-H_mut_-αCD20 encoding the truncated H_mut_ protein displaying the αCD20-scFv was described previously [7]. Briefly, the αCD20-scFv was linked to the ectodomain of H_mut_ protein via the factor Xa cleavage site (Xa). To generate the plasmids pCG-H_mut_-αCD19 and pCG-H_mut_-αCD19ds, the respective αCD19-scFv coding regions were PCR amplified with the primers CD19-SfiI+ (TGCTTGGCCCAGCCGGCCATGGCGGACTACAAAGATATTG) and CD19-NotI- (TATTCCTTTTGCGGCCGCCGAGGAGACGGTGACTGTG) using pSecTag2HygroC-CD19-4G7-EGFP [24] and pSecTag2HygroC-Strep-CD19-4G7dsxCD16ds [23] as templates encoding either the unstabilized or the disulfide bond stabilized CD19-specific scFv which was subcloned from the hybridoma clone 4G7 [23], [24], [41]. The PCR amplificates were digested with SfiI and NotI and ligated into the SfiI and NotI digested pCG-H_mut_L3 backbone encoding the cytoplasmic tail-truncated H_mut_ protein with a (G_4_S)_3_ linker at its C-terminus [13].

The H protein plasmids were purified with the EndoFree® Plasmid Maxi Kit (Qiagen, Hilden, Germany), whereas all other plasmids used for vector particle production were purified by the PlasmidFactory GmbH & Co. KG, Bielefeld, Germany.

### Cultivation of Cell Lines and Primary Human B Lymphocytes

Culture of HEK-293T cells, Raji cells and Molt 4.8 cells has been described previously [7], [16]. Primary human B lymphocytes were isolated from fresh human peripheral blood mononuclear cells (PBMCs) using the Dynal B cell negative isolation kit (Invitrogen, Karlsruhe, Germany) and cultivated in RPMI 1640 medium supplemented with 10% FCS, 1% glutamine, 0.5% streptomycin/penicillin and 25 mmol/l HEPES [7]. The PBMCs were either obtained from buffy coats by centrifugation over Histopaque-1077 (Sigma-Aldrich, Taufkirchen, Germany) or for the cell cycle analysis experiments from donors using the BD Vacutainer™ CPT™ system with sodium citrate (BD, Heidelberg, Germany). Immediately after isolation, the purity of isolated B lymphocytes was monitored by FACS analysis using PE-conjugated anti-CD19 antibody (DakoCytomation, Glostrup, Denmark) and PE-Cy5-conjugated anti-CD20 antibody (BD Pharmingen, Heidelberg, Germany). Unstimulated B lymphocytes were confirmed to be negative for the activation marker CD69 using the PE-conjugated anti-CD69 antibody (BioLegend, San Diego, USA).

### Vector Particle Production and Titration

Lentiviral vector particles were produced by polyethyleneimine (PEI) based transfection of HEK-293T cells as described previously [42]. Briefly, 2×10^7^ cells were seeded 24 h before transfection into a T175 flask. On the day of transfection, medium was replaced with 10 ml of DMEM supplemented with 15% FCS and 2 mM L-glutamine. For targeting vectors, 1.35 µg of pCG-H_mut_-αCD20 [7] or pCG-H_mut_-αCD19ds, 4 µg of pCG-FcΔ30 [7], 14.4 µg of packaging plasmid pCMVΔR8.9 [43] and 15.2 µg of transfer vector plasmid pSEW [44], encoding EGFP, were mixed with 2.3 ml DMEM without supplements. For MV-LV vectors, 1.35 µg of pCG-HcΔ18 [7] and 9.4 µg of pCG-FcΔ30 together with 9.1 µg pCMVΔR8.9 and 15.2 µg pSEW were applied. In parallel, 140 µl of 18 mM PEI and 2.2 ml DMEM without supplements were mixed. The PEI mixture and the DNA mixture were combined, vortexed and incubated for 20 min at room temperature (25°C), before the transfection mixture was added to the cells. Twenty-four hours later, medium was exchanged against 16 ml fresh DMEM supplemented with 10% FCS and 2 mM L-glutamine. The cell supernatant containing the pseudotyped vector particles was filtered 24 h later (0.45-µm filter). The vector particles were concentrated 270-fold by ultracentrifugation over a 20% (wt/vol) sucrose cushion (100,000 g for 3 h at 4°C). Vector particles pseudotyped with VSVG were produced by co-transfection of 6.13 µg pMD.G2 (kindly provided by Didier Trono, Tronolab, Lausanne, Switzerland), 11.4 µg pCMVΔR8.9 and 17.5 µg pSEW. Targeting vectors and control vectors were titrated on Raji cells. EGFP^+^ cells were determined by FACS analysis and vector stock titers were calculated as described previously [7], [13]. Briefly, cells were transduced by at least five serial dilutions of vector particles. Titer calculation was based on those samples showing a linear correlation of EGFP expression with the dilution factor. We observed correlations in the range from four-fold up to nine-fold increase in transduction efficiency by a ten-fold increase in supernatant volume for all lentiviral vectors.

### Transduction of Cells

For transduction of cell lines, 1×10^5^ cells per well of a 48 well plate were incubated with the indicated amount of vector particles and 4 µg/ml protamine sulfate (Sigma-Aldrich, Munich, Germany) in a final volume of 250 µl for 3 h at 37°C in a cell culture incubator. Then, 500 µl fresh medium was added. After 72 h, transgene expression was determined by FACS analysis. To study specific gene transfer, Raji and Molt 4.8 cells were co-cultivated at the indicated ratios and transduced by CD19ds-LV or VSVG-LV vectors at an MOI of 2 calculated based on total cell numbers. For transduction of freshly isolated B lymphocytes, the indicated vector particles and 4 µg/ml protamine-sulfate were added together with 3×10^4^ B lymphocytes in a final volume of 100 µl into a well of a 96 well plate and were centrifugated for 1 h at 480×g, 32°C. Then, 100 µl fresh medium was added and the cells were cultured for 48 h at 37°C in a cell culture incubator, before the percentage of EGFP^+^ B lymphocytes was determined by FACS analysis. For long-term culture of transduced unstimulated B lymphocytes, the cells were transferred onto MS-5 feeder cells [45] in RPMI supplemented with 10% FCS, 10 ng/ml rhIL-15 (Roche, Mannheim, Germany) and 10 ng/ml rhIL-2 (Roche, Mannheim, Germany) and passed onto a new MS-5 cell monolayer every 4 days.

### Flow Cytometry

Flow cytometry and identification of B lymphocytes was performed as described previously [13]. For surface expression experiments, 2×10^5^ HEK-293T cells were transfected in a 6 well plate with pCG-H_mut_-αCD19, pCG-H_mut_-αCD19ds and pCG-1 expression plasmids, respectively, by Lipofectamine transfection reagent (Invitrogen, Darmstadt, Germany) according to the manufacturer’s instructions. After 48 h, cells were detached by PBS-trypsin incubation and washed once in FACS washing buffer (PBS, 1% FCS, 0.1% NaN_3_) before they were incubated for 20 min at 4°C in the dark with a PE-conjugated anti-His antibody (MiltenyiBiotec, Bergisch Gladbach, Germany) to detect His-tagged H_mut_-αCD19 and H_mut_-αCD19ds proteins. After antibody incubation, the cells were washed twice in FACS washing buffer and were then fixed in 100 µl PBS/1% paraformaldehyde, before they were applied to FACS analysis. For detection of CD8^+^ Molt 4.8 cells in cell mixtures, an APC-conjugated anti-CD8 antibody (BD Pharmingen, Heidelberg, Germany) was used. For detection of CD19^+^/CD20^+^ B lymphocytes, a PE-conjugated anti-CD19 antibody (DakoCytomation, Glostrup, Denmark) and a PE-Cy5-conjugated anti-CD20 antibody (BD Pharmingen, Heidelberg, Germany) was applied. To identify B lymphocyte activation, PE-conjugated anti-CD69 antibody (BioLegend, San Diego, USA) and PE-conjugated anti-CD71 antibody (BD Pharmingen, Heidelberg, Germany) was used. The respective isotype control antibodies were applied as controls. Data were obtained with the LSR II FACS machine (Becton Dickinson, Heidelberg, Germany) and analyzed with the FACSDiva software (Becton Dickinson, Heidelberg, Germany) or the FCS express software (De novo Software, Los Angeles, CA, USA).

### Cell Cycle Analysis

Cell cycle analysis was performed by staining the DNA and RNA content of B lymphocytes with 7-Aminoactinomycin D (7-AAD; Sigma-Aldrich, Munich, Germany) and pyronin Y (Sigma-Aldrich, Munich, Germany), respectively. 1×10^5^ cells were washed with 4 ml PBS before they were incubated for 30 min at room temperature (25°C) in 300 µl NASS buffer (0.1 M phosphate-citrate, 5 mM EDTA, 0.5% w/v BSA, 0.15 M NaCl, 0.03% Saponine, pH 6.0 in H_2_O) supplemented with 0.02 µg/µl 7-AAD. After 5 min incubation on ice, 0.007 µg/µl pyronin Y was added. The cells were incubated for additional 10 min on ice, before they were immediately analyzed on a LSR II FACS machine.

### Calcium Assay

Freshly isolated primary human B lymphocytes were incubated in serum-free, calcium-free PBS containing 5 µM Calcium Sensor Dye eFluor® 514 (eBioscience, Frankfurt, Germany) for 30 minutes at 37°C. Cells were then washed twice with PBS and resuspended at 5×10^5^ cells/mL in HBSS (Hank’s Balanced Salt Solution) (Invitrogen, Darmstadt, Germany) supplemented with 20 mM HEPES (N-2-hydroxyethylpiperazine-N9-2-ethanesulfonic acid) (Invitrogen, Darmstadt, Germany). The viability of cells was higher than 95% as determined by trypan blue dye exclusion. The cells were kept at room temperature until they were analyzed by FACS using a LSR II flow cytometer (Becton Dickinson, Heidelberg, Germany). For each sample, a 60 s baseline monitoring was performed. Then sample aspiration was briefly paused and stimulant (CD20-VLP, CD19ds-VLP, VSVG-VLP or ionomycine) was quickly added before the acquisition was continued at rates of approximately 300 events/second for a total of 250 s. The changes in calcium concentration were analyzed by the change in mean fluorescent intensity (MFI) of eFluor® 514-labeled cells over the time. The data acquired were analyzed using FCS Express software (De novo Software, Los Angeles, CA, USA).
